# Gain-Framed Text Messages and Nicotine Replacement Therapy for Smoking Cessation Among Lung Cancer Screening Patients: A Brief Report of a Pilot Randomized Controlled Trial

**DOI:** 10.3390/ijerph22040543

**Published:** 2025-04-02

**Authors:** Kinsey Pebley, Benjamin A. Toll, Matthew J. Carpenter, Gerard Silvestri, Alana M. Rojewski

**Affiliations:** 1 Department of Public Health Sciences, Medical University of South Carolina, 135 Cannon St., Charleston, SC 29425, USA; pebley@musc.edu (K.P.); toll@musc.edu (B.A.T.); 2 Hollings Cancer Center, Medical University of South Carolina, 86 Jonathan Lucas St., Charleston, SC 29425, USA; carpente@musc.edu (M.J.C.); silvestr@musc.edu (G.S.); 3 Department of Psychiatry and Behavioral Sciences, Medical University of South Carolina, 67 President St., Charleston, SC 29425, USA; 4 Division of Pulmonary, Critical Care and Sleep Medicine, Medical University of South Carolina, 135 Rutledge Ave., Charleston, SC 29425, USA

**Keywords:** cancer, lung cancer, lung cancer screening, smoking, smoking cessation, tobacco, tobacco cessation treatment, nicotine, nicotine treatment, text messaging

## Abstract

People who undergo lung cancer screening (LCS) and continue to smoke are at risk for negative clinical outcomes and lowered survival and need effective smoking cessation interventions. This pilot study tested an 8-week intervention for smoking cessation after LCS. The participants (*N* = 40) were randomized to the intervention group (combination nicotine replacement therapy [NRT] plus gain-framed text messaging for 8 weeks) or the control group (standard cessation counseling) after LCS. Assessments were completed at 8-week and 3-month follow-ups, including self-reported 7-day point prevalence abstinence. The mean age was 64.4 years old (SD = 6.2); 32.5% were Black or African American; and 55% were female. At Week 8, 14.3% (3/21) of the participants in the intervention group were abstinent versus 0% (0/19) in the control group (*p* > 0.05). At 3-month follow-up, 4.8% (1/21) of the participants in the intervention group were abstinent versus 0% (0/19) in the control group. Among the intervention group participants, up to 52.4% used the provided patches and up to 61.9% used the provided lozenges during the study period. This study demonstrated modest quit rates for LCS patients receiving gain-framed text messages and NRT. The results highlight the need for more effective smoking cessation interventions for this priority population.

## 1. Introduction

Lung cancer screening (LCS) with low-dose computed tomography (LDCT) affords identification of lung cancer at earlier stages in high-risk patients, particularly those who smoke [1]. Patients who engage in screening and achieve smoking cessation have a 38% reduction in lung cancer mortality [1]. However, effective cessation interventions are lacking for this population. Recent trial results in this area (i.e., from the Smoking Cessation at Lung Examination [SCALE] Collaboration) [2] indicate that the tested interventions did not yield sustained cessation rates compared with controls [3,4,5,6]. Thus, there is still a need for tobacco intervention development among LCS patients.

Many patients face barriers to accessing existing smoking cessation treatments [7]. Text messaging may extend access, and evidence shows text message-based smoking cessation programs are effective [8]. Message framing may also influence behavior. Prospect theory [9] suggests decision-making under conditions of risk is influenced by message framing. Individuals evaluate information relevant to decisions in terms of potential gains (i.e., benefits) or potential losses (i.e., costs) compared with a reference point, with gain-framed messaging being more effective than loss-framed messaging for increasing intentions to quit smoking, quit attempts, and smoking cessation in the general population [10,11,12]. Additionally, nicotine replacement therapy (NRT) may be helpful in promoting smoking cessation among LCS patients, as it has been shown to increase the likelihood of making a quit attempt and of successful cessation [13,14]. Tobacco cessation treatment increases when accessibility is increased, and providing NRT to LCS patients may increase this accessibility [15]. Thus, using gain-framed text messaging with pharmacotherapy may be an accessible, effective approach to facilitating smoking cessation among LCS patients.

The current pilot parallel randomized controlled trial tested an 8-week tobacco cessation treatment intervention using gain-framed text messages and nicotine replacement therapy (NRT) for LCS patients. This study’s aims included assessing abstinence rates at the end of treatment and follow-up to generate effect size estimates.

## 2. Materials and Methods

### 2.1. Participants and Procedures

Potential participants were identified from 2019 to 2023 through the Medical University of South Carolina’s (MUSC) Hollings Cancer Center LCS program in South Carolina, United States. The eligible participants met the criteria for LCS in the U.S.: age 55+ years, currently smoking, and 30+ pack year history of smoking. The United States Preventive Services Task Force LCS criteria were updated in 2021, and the inclusion criteria were changed to reflect these new guidelines: age 50+ years and 20+ pack year smoking history. Additionally, the participants needed to speak English and have access to a device with text messaging. Individuals were excluded if they had unstable psychiatric/medical conditions (acute suicidality, acute psychosis, severe alcohol dependence), certain cardiovascular conditions (myocardial infarction in the past two weeks, unstable angina pectoris, serious arrythmias, or hemodynamically or electrically unstable), or were already receiving tobacco cessation care elsewhere. Informed consent was obtained from all the participants involved in this study. The study procedures were approved by the MUSC Institutional Review Board, and this study was registered at clinicaltrials.gov (NCT03069924).

Patients who completed an LCS shared decision-making visit and an LDCT scan were contacted within 30 days following their visit; the interested patients were screened, consented, and randomized 1:1 using block randomization to the control or the intervention group by the study staff. All the participants had a brief smoking cessation counseling session at the time of their shared decision-making visit with the nurse practitioners (cross-trained as Tobacco Treatment Specialists) [16], as is standard clinical care. All the participants completed assessments at baseline, 8-weeks post-randomization (end of treatment for intervention group), and 3 months after treatment completion. Medication use and smoking frequency were also assessed every two weeks during the treatment period. The control group participants were not precluded from seeking tobacco cessation treatment.

The intervention group participants received 2 weeks of combination NRT (21 mg patches and 4 mg lozenges), and 3 additional 2-week supplies of combination NRT were mailed throughout the 8-week intervention upon request. The participants were also sent three gain-framed text messages per day for 8 weeks. Specific messages were tailored to the context of LCS and their scan results (e.g., whether a nodule was found or not), while others were general (e.g., encouraging patch use). Some messages were interactive and encouraged a response from participants (“It has been 4 weeks since we saw you for lung screening. Are you smoke free or still smoking? Reply with FREE or SMOKE”.), while others were informational and supportive. The messages were sent via Twilio, which paired with the REDCap system used for data management to time the sending of text messages after the LCS scan was completed and the results were known.

Baseline survey questions assessed demographics, smoking behaviors, and current medication use. Brief surveys every two weeks inquired about smoking status and medications. Follow-up surveys at 8 weeks and 3 months assessed medication use by providing a medication list and asking whether the participants had used any (yes/no) since the last assessment. If the participants endorsed the use of a medication, they were asked to recall how many units of that product they used on each day since their last assessment (e.g., number of lozenges per day). A timeline follow-back [17] was also completed at the Week-8 and 3-month follow-ups. The seven-day point prevalence abstinence from smoking, the primary outcome, was determined from this measure. The participants were provided with iCOquit Smokerlyzers, which are devices that measure exhaled carbon monoxide and serve as a biochemical verification technique for smoking cessation. No participants completed carbon monoxide testing within the specified follow-up timeframe, and, thus, these results were not used in the analyses. The intervention group participants also answered questions about text message satisfaction at follow-up. All the participants were compensated with USD 10 Amazon giftcard per survey for completing the two-week surveys and received an additional USD 30 gift card if they completed 3 of the 4 two-week surveys. The participants also received USD 25 giftcard for completing the 3-month follow-up survey.

### 2.2. Statistical Analysis

A sample size of 80 was initially selected as this would allow for effect size estimates of abstinence with 95% confidence intervals for each arm. However, the final sample size was reduced due to the COVID-19 pandemic, which interrupted recruitment. A total of 48 individuals were enrolled into this study, but 8 withdrew after initial consent. Thus, the final sample was comprised of 40 participants. Descriptive statistics were used to characterize the sample and calculate Week 8 and 3-month abstinence rates. Individuals with missing data related to smoking behaviors were coded as smoking for the intent-to-treat analysis. Chi-square tests were used to determine whether abstinence rates were significantly different between groups at Week 8 and 3 months. The analyses were conducted using SPSS version 25.

## 3. Results

**Figure 1:**
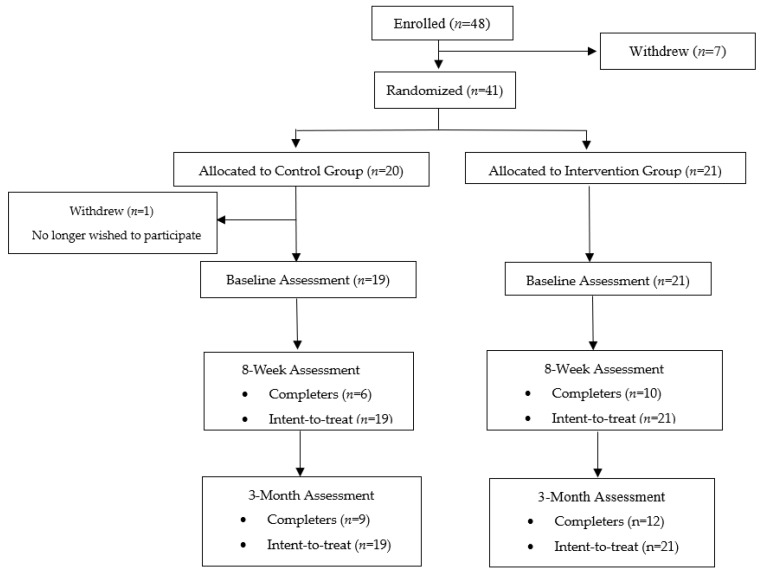
CONSORT diagram.

Table 1 displays the sample characteristics, and the CONSORT diagram is presented in Figure 1. The mean age of the participants (*N* = 40) was 64.4 years old (standard deviation = 6.2); 32.5% were Black or African American; 55% were female; and the mean number of cigarettes smoked per day at baseline was 11.8 in the control group and 17.6 in the intervention group. The number of cigarettes smoked per day was significantly different between the intervention and control groups at baseline (t(df) = −2.1, *p* = 0.04). However, the nicotine dependence scores were not significantly different. All the other demographic characteristics were not statistically different between groups (*p* > 0.05). At Week 8, 14.3% *(n* = 3/21) of the intervention group participants were abstinent versus 0% *(n* = 0/19) of the control group participants (χ^2^ = 2.55, *p* = 0.11); the 3-month follow-up rates were 4.8% *(n* = 1/21; sustained from Week 8) versus 0% *(n* = 0/19) in the control group (χ = 0.14, *p* = 0.71).

Table 2 presents medication use data by group. All medications for the control participants and medications other than lozenges or patches for the intervention participants were acquired outside of this study. Medication use was limited in the control group. Among the intervention group participants, patch use ranged from 38.1 to 52.4% and lozenge use ranged from 19.0 to 61.9% across the intervention and follow-up periods. Participants within the intervention group could request refills of NRT as part of their two-week inquiries (2-, 4-, and 6-weeks post-randomization). At two weeks, 28.6% requested additional patches and lozenges; 14.3% requested additional patches; and 4.8% requested additional lozenges. At four weeks, 19.0% requested additional patches and lozenges, and 14.3% requested additional patches. At six weeks, 23.8% requested additional patches and lozenges, and 4.8% requested additional lozenges.

In relation to the intervention group participants’ satisfaction with text messages, all the participants reported the texts were easy to understand. Most reported that they were moderately to extremely satisfied with the texts (90%), that the messages were helpful for quitting smoking (90%) and personalized to them (70%), and that three messages were the “perfect amount” (90%). Ten intervention participants (47.6%) responded to interactive intervention text messages. Table 3 displays the text message satisfaction information.

## 4. Discussion

The current study tested a tailored text messaging and medication tobacco cessation treatment intervention for LCS patients. While quit rates were low and not significantly different between groups, there was a signal to suggest benefit from the intervention. At Week 8, 14.3% of intervention group participants had quit smoking, and 4.8% of participants sustained abstinence at the 3-month follow-up. This rate is lower than the quit rates reported in the four SCALE collaborative trials (13.0–17.8%) published to date [3,4,5,6]. However, some SCALE trials had different definitions of abstinence (prolonged abstinence, using smoking status changes in the medical record) [4,6] and had different follow-up periods (i.e., 6 months, 12 months) [3,4,6]. The SCALE trial assessing seven-day point prevalence at three months (similar to the current study’s primary outcome) reported an abstinence rate of 14.3% [5]. The current intervention may not be the optimal choice for LCS patients. This population may require a more intensive intervention to facilitate cessation, as the current intervention was remote and less intensive than those in the four published SCALE trials, which may enhance scalability and increase the reach of interventions but have lower efficacy.

Only up to 52.5% of the participants in the intervention group used the provided nicotine patches and up to 61.9% used the provided nicotine lozenges, despite this NRT being proactively provided at no cost. It may be important to consider in future trials how to increase medication uptake and to ask the participants who decline medications about their reasons for declination. There may be some misperceptions about NRT, as previous studies have demonstrated individuals are often not accurately informed about the impact of nicotine on health and the harm reduction associated with NRT [18,19]. Thus, addressing these misconceptions and providing detailed instructions for use may be prudent for LCS patients seeking to quit smoking.

The current study has some limitations, including a small sample size. The premise of this study was to generate effect size estimates for a future fully powered clinical trial, and this study experienced difficulty in recruitment due to COVID-19. Second, there was no method available to confirm that the text messages were received or read or to determine how long it took for the participants to respond to interactive text messages. Thus, there may have been unknown delivery or technological errors. Twilio was used in the current study given its compatibility with REDCap. However, it may be that other HIPAA-compliant messaging systems that provide additional metrics (e.g., information about message delivery) are needed to conduct high-quality research employing text messaging. Additionally, other digital health tools such as mobile apps may be appealing to individuals undergoing LCS given the rise in popularity of these resources. Lastly, we relied on the self-reported smoking status. Carbon monoxide measurements were to be collected among individuals reporting seven-day abstinence using a device mailed to participants, but no participants were able to complete the test within the specified timeframe.

Overall, the current study provides insight into smoking cessation interventions among individuals undergoing LCS and provides some lessons learned. Obtaining carbon monoxide biochemical verification was challenging, and this population may need additional support to employ such measures. Additionally, the results indicated that tailored text messages were well received and may be acceptable to implement in other interventions among LCS patients. Lastly, technological considerations are important when selecting a platform to use, including using text messaging platforms that provide metrics related to whether messages are received or read, as these can promote high-quality research and enable investigators to assess the levels of engagement with such an intervention.

## 5. Conclusions

The current intervention using NRT and gain-framed, tailored text messages produced modest abstinence rates at Week 8. However, the rates were not significantly different from those for the control group. These findings contribute to the literature on smoking cessation efforts in people undergoing LCS and suggest that more work is needed to develop effective interventions for these patients.

## Figures and Tables

**Table 1:** Demographic characteristics.

Characteristic	Overall Sample (*N* = 40)	Intervention Group (*n* = 21)	Control Group (*n* = 19)
	*N* (%)	*N* (%)	*N* (%)
**Insurance Status ^a^**			
Medicare	17 (42.5%)	10 (47.6%)	7 (36.8%)
Medicaid	14 (35.0%)	7 (33.3%)	7 (36.8%)
Private	14 (35.0%)	8 (38.1%)	6 (31.6%)
None	1 (2.5%)	0 (0%)	1 (5.3%)
Other	4 (10.0%)	1 (4.8%)	3 (15.8%)
**Sex**			
Female	22 (55%)	12 (57.1%)	10 (52.6%)
Male	18 (45%)	9 (42.9%)	9 (47.4%)
**Marital Status**			
Single	7 (17.5%)	2 (9.5%)	5 (26.3%)
Married	18 (45.0%)	11 (52.4%)	7 (36.8%)
Separated	1 (2.5%)	1 (4.8%)	0 (0%)
Divorced	9 (22.5%)	5 (23.8%)	4 (21.1%)
Widow	4 (10.0%)	1 (4.8%)	3 (15.8%)
Cohabitating	1 (2.5%)	1 (4.8%)	0 (0%)
**Race ^a^**			
Black or African American	13 (32.5%)	5 (23.8%)	8 (42.1%)
White	27 (67.5%)	15 (71.4%)	12 (63.2%)
Other	1 (2.5%)	1 (4.8%)	0 (0%)
**Ethnicity**			
Non-Hispanic	40 (100.0%)	40 (100%)	40 (100%)
**Education Level**			
Less than high school/GED	10 (25.0%)	4 (19.1%)	6 (31.6%)
High school diploma/GED	13 (32.5%)	6 (28.6%)	7 (36.8%)
Vocational training	3 (7.5%)	2 (9.5%)	1 (5.3%)
Associate’s degree/Some college	11 (27.5%)	8 (38.1%)	3 (15.8%)
Bachelor’s degree or higher	3 (7.5%)	1 (4.8%)	2 (10.6%)
**Employment Status**			
Employed full-time	5 (12.5%)	2 (9.5%)	3 (15.8%)
Employed part-time	6 (15.0%)	2 (9.5%)	4 (21.1%)
Unemployed or disabled	18 (45.0%)	9 (42.9%)	9 (47.4%)
Retired	10 (25.0%)	8 (38.1%)	2 (10.5%)
Other	1 (2.5%)	0 (0%)	1 (5.3%)
**Income (amount per year)**			
<USD 8000–USD14,999	11 (27.5%)	5 (23.8%)	6 (31.6%)
USD 15,000–USD 24,999	8 (20.0%)	4 (19.0%)	4 (21.1%)
USD 25,000–USD 34,999	5 (12.5%)	1 (4.8%)	4 (21.1%)
USD 35,000–USD 49,999	6 (15.0%)	3 (14.3%)	3 (15.8%)
USD 50,000–USD 64,999	3 (7.5%)	3 (14.3%)	0 (0%)
USD 65,000–USD 79,999	1 (2.5%)	0 (0%)	1 (5.3%)
USD 80,000–USD 100,000	1 (2.5%)	1 (4.8%)	0 (0%)
>USD 100,000	3 (7.5%)	3 (14.3%)	0 (0%)
**Age in years (Mean, SD)**	64.4 (6.2)	65.4 (6.1)	63.4 (6.3)
**Nicotine dependence (FTND Score; Mean, SD)**	4.3 (1.9)	4.1 (2.1)	4.4 (1.7)
**Cigarettes per day at baseline (Mean, SD)**	14.8 (9.0)	17.6 (9.42)	11.8 (7.6)

Note: a = participants could select more than one option, which may result in a percentage total higher than 100% in that category; SD = standard deviation.

**Table 2:** Number of participants reporting medication use over time across groups.

Nicotine Replacement Therapy Product	2 Weeks *n* (%)	4 Weeks *n* (%)	6 Weeks *n* (%)	8 Weeks *n* (%)	12 Weeks
**Intervention Group**					
Patches	9 (42.9%)	10 (47.6%)	11 (52.4%)	9 (42.9%)	8 (38.1%)
Lozenges	13 (61.9%)	9 (42.9%)	8 (38.1%)	4 (19.0%)	4 (19.0%)
Gum	2 (9.5%)	1 (4.8%)	0 (0%)	0 (0%)	0 (0%)
Nasal spray	1 (4.8%)	0 (0%)	0 (0%)	0 (0%)	0 (0%)
**Control Group**					
Patches	0 (0%)	0 (0%)	0 (0%)	0 (0%)	1 (4.8%)
Lozenges	0 (0%)	0 (0%)	0 (0%)	0 (0%)	0 (0%)
Gum	0 (0%)	0 (0%)	1 (4.8%)	0 (0%)	0 (0%)
Nasal spray	0 (0%)	1 (4.8%)	0 (0%)	0 (0%)	0 (0%)

Note: Individuals who did not respond to a follow-up survey were considered to not be using a nicotine replacement therapy product. The values reported reflect individuals who reported any amount of product use since the previous assessment at that time period.

**Table 3:** Satisfaction with text messages among intervention group participants.

Question	*N* (%)
**The advice that I received via text was easy to understand.**	
Strongly disagree	0 (0%)
Disagree	0 (0%)
Somewhat disagree	0 (0%)
Neither agree nor disagree	0 (0%)
Somewhat agree	0 (0%)
Agree	6 (60.0%)
Strongly agree	4 (40.0%)
**The text messages were helpful in my attempt to quit smoking.**	
Strongly disagree	0 (0%)
Disagree	1 (10.0%)
Somewhat disagree	0 (0%)
Neither agree nor disagree	0 (0%)
Somewhat agree	0 (0%)
Agree	7 (70.0%)
Strongly agree	2 (20.0%)
**I felt the texts were personalized to me.**	
Strongly disagree	1 (10.0%)
Disagree	2 (20.0%)
Somewhat disagree	0 (0%)
Neither agree nor disagree	0 (0%)
Somewhat agree	0 (0%)
Agree	6 (60.0%)
Strongly agree	1 (10.0%)
**You received 3 text messages per day. Did you think that this was…**	
Far too few texts	0 (0%)
Too few texts	0 (0%)
The perfect amount of texts	9 (90.0%)
Too many texts	1 (10.0%)
Far too many texts	0 (0%)
**Were you satisfied with the interactive texts?**	
Not at all satisfied	1 (10.0%)
Slightly satisfied	0 (0%)
Moderately satisfied	1 (10.0%)
Very satisfied	4 (40.0%)
Extremely satisfied	4 (40.0%)

Note: 10 of 21 participants in the intervention group answered these questions. The denominator for calculating percentages is 10.

## Data Availability

Data will be made available upon reasonable request. Data use agreements may need to be signed, as appropriate.
